# Liver Transplantation as a Definitive Treatment for Homozygous Protein C Deficiency

**DOI:** 10.7759/cureus.78498

**Published:** 2025-02-04

**Authors:** Juwairiya Syed Iqbaluddin, Saista Asif Amin, Hanan Fawzy Nazir Abouelkhel, Johns Shaji Mathew, Gourab Sen, Rehan Saif

**Affiliations:** 1 Pediatrics, Al Qassimi Women's and Children's Hospital, Sharjah, ARE; 2 Pediatric Gastroenterology, American Hospital Dubai, Dubai, ARE; 3 Pediatric Hematology, Al Qassimi Women's and Children's Hospital, Sharjah, ARE; 4 Hepatobiliary and Abdominal Multi-organ Transplant Surgery, Burjeel Medical City, Abu Dhabi, ARE

**Keywords:** fresh frozen plasma, homozygous protein c deficiency, liver transplantation, purpura fulminans, retinal haemorrhage

## Abstract

Protein C is a vitamin K-dependent anticoagulant glycoprotein that inhibits activated factors V and VIII once activated by thrombin while promoting fibrinolysis. Patients with homozygous protein C deficiency generally present shortly after birth with purpura fulminans, retinal hemorrhage, and signs of the central nervous system or renal thrombosis. This case reports the first case of homozygous protein C deficiency in the UAE successfully treated with liver transplantation. This article aims to emphasize the importance of early diagnosis of neonatal purpura fulminans based on clinical presentation and to maintain a high index of suspicion for protein C deficiency. It seeks to illustrate that liver transplantation can effectively restore protein C activity, offering a life-saving treatment for a condition that would otherwise be devastating. Additionally, in resource-limited settings, the article highlights the potential use of fresh frozen plasma (FFP) as an alternative to protein C concentrate for supporting protein C levels during the perioperative period. We present an eight-year-old female who was diagnosed with protein C deficiency at birth in her home country following the onset of purpura fulminans. She received regular FFP transfusions but continued to experience complications such as blindness, leg contractures, and infections despite treatments with protein C concentrate and warfarin. Seeking further care, she was evaluated in the UAE for liver transplantation, a potentially curative treatment for this disorder. After being placed on the national transplant waiting list, the patient underwent a successful liver transplantation from a deceased donor. Perioperative management included careful monitoring of protein C levels and blood products to prevent thrombotic and bleeding complications. Her protein C activity normalized post-transplant, and there were no thrombotic events. This case highlights the potential of liver transplantation as a definitive cure for homozygous protein C deficiency. In conclusion, liver transplantation should be considered as a definitive treatment for children with homozygous protein C deficiency who experience recurrent thrombotic events despite replacement therapy and for those with severely impaired quality of life due to the disease itself. The decision to proceed with transplantation should be made with careful consideration of the patient’s long-term quality of life and the balance between transplant-related risks and the benefits of avoiding continuous anticoagulation therapy.

## Introduction

Protein C is a vitamin K-dependent anticoagulant glycoprotein that inhibits activated factors V and VIII once activated by thrombin while promoting fibrinolysis. Evidence indicates that protein C deficiency follows an autosomal dominant inheritance pattern. Individuals heterozygous for protein C deficiency typically exhibit around 50% of normal protein C activity. Individuals with homozygous protein C deficiency usually show symptoms shortly after birth, including purpura fulminans, retinal hemorrhage, and signs of central nervous system or renal thrombosis. In all reported cases, protein C activity levels are undetectable, and the condition is invariably fatal without treatment [1].

The conventional management includes protein C replacement therapy using fresh frozen plasma (FFP) or protein C concentrates to maintain adequate protein levels and prevent thrombotic complications. Long-term management, however, can be challenging due to the need for frequent treatments and the risk of infection, thrombosis, or allergic reactions. Hepatic transplantation offers a potential curative treatment for homozygous protein C deficiency. The liver is the primary site of protein C synthesis, and successful liver transplantation can restore normal protein C levels, eliminating the need for lifelong replacement therapy.

This case report presents the first pediatric patient with homozygous protein C deficiency who was successfully treated with hepatic transplantation in the UAE, highlighting the potential of this intervention as a definitive cure for this rare and severe disorder.

## Case presentation

An eight-year-old female with a known case of homozygous protein C deficiency presented to our hospital for regular FFP transfusions. She was born at term in her home country via lower segment cesarean section, which was performed due to decreased fetal movements. There were no antenatal complications or any complications during delivery. She did not require admission to the neonatal intensive care unit. Neonatal screening was not performed. The parents of the patient are cousins, and the family history is remarkable of two sibling deaths; one passed away in utero, while another died at eight months of age secondary to a bacterial infection. The patient has one healthy sister, and there are no known hereditary chronic conditions in the family. She is not on any chronic medications and has no known allergies. Her developmental milestones are appropriate for her age, and her immunization status is current.

Our patient was diagnosed with protein C deficiency following the onset of purpura fulminans on day 2 of life, which appeared as necrotic lesions over her back and extremities. Significantly low protein C levels confirmed her diagnosis. Since infancy, she has been maintained on FFP transfusions every other day to manage her protein C deficiency.

She was further started on protein C concentrate and warfarin; however, these treatments were discontinued for social and logistical reasons. Despite different attempts at management, she developed several complications. During the neonatal period, she required surgical intervention on her left leg for nerve damage, which involved skin grafting. In addition, she had developed blindness due to a retinal hemorrhage, a complication of her condition. At the age of three years, she underwent another surgery for tendon release, which arose secondary to an extravasation injury during cannula insertion in early infancy. Furthermore, she experienced two episodes of pseudomonas port-a-Cath infections at the ages of two and four years, both of which required hospital admission and treatment with intravenous antibiotics for 14-21 days.

For the past several years, she has been relying on FFP transfusions. Her family reports multiple episodes of skin rash and shortness of breath after FFP administration. She and her family recently came to the UAE to seek a second opinion regarding her ongoing care and management.

Before liver transplantation, basic investigations revealed normal complete blood count parameters, with hemoglobin at 11.6 g/dL and a normal platelet count. Renal function tests and liver function tests were also within normal ranges. Inflammatory markers were not elevated. The coagulation profile was normal, with a prothrombin time of 12.8 seconds, an activated partial thromboplastin time of 30.8 seconds, and an international normalized ratio of 1.08. However, protein C activity was significantly low at 5%.

She had an ultrasound Doppler of the carotid and femoral vessels done at six years of age, which revealed normal caliber color coding and flow direction in all vessels. The right jugular vein exhibited increased wall thickening with a stenotic segment distally, while the color coding and flow direction appeared normal. The remainder of the study was unremarkable. Common femoral and superficial femoral veins revealed compressible walls, no thrombus was seen bilaterally, and there was a mild increase in wall thickening at the left superficial femoral vein. No evidence of deep vein thrombosis.

The patient required a multidisciplinary management approach involving the general pediatric team, gastroenterologist/hepatologist, hematologist, and liver transplantation team. After discussion, it was concluded that liver transplantation would offer a definitive cure. A comprehensive pre-transplant evaluation was conducted to confirm her suitability for surgery, and she was found fit for transplantation. The option of living donor liver transplantation was excluded due to the lack of a compatible donor within her family. Consequently, she was placed on the national transplant waiting list. After approximately two months on the waiting list, the patient received an organ offer through the national allocation system. The donor, a female with a petite build, matched the recipient’s height and weight.

To maintain protein C levels, she was initiated on FFP transfusions at a dosage of 20 mL/kg to be given over 30 minutes twice a week. Additionally, her trough protein C levels were closely monitored to remain above 25%.

Perioperative management of blood products in liver transplantation is inherently challenging, especially given the risks associated with FFP administration. While FFP is commonly used pre-transplant as a source of protein C, its perioperative use can lead to vascular complications in the small vessels requiring reconstruction. Discontinuation of FFP, however, poses a significant risk of thrombotic complications due to the newly transplanted liver's delayed onset of protein C synthesis. To mitigate these risks, therapeutic doses of low molecular weight heparin were administered in the immediate perioperative period, supplemented with protein C concentrate (Ceprotin) during the first few days post-transplant.

On postoperative day three, the patient exhibited active bleeding from the surgical drain, necessitating an exploratory reoperation. The source was identified as diffuse oozing from the muscle layer, likely secondary to elevated enoxaparin levels. Following the intervention, the enoxaparin dosage was reduced, and protein C activity was monitored until it reached a target level of >100%, at which point Ceprotin was discontinued.

The patient’s immunosuppressive regimen consisted of standard steroid-based induction, followed by maintenance therapy with tacrolimus, mycophenolate mofetil, and prednisolone. Tacrolimus trough levels were closely monitored and maintained at 5 ng/mL through thrice weekly assays with doses adjusted as required to ensure therapeutic levels. Ultrasound of the abdomen and liver Doppler post-transplant was normal. There were no thrombotic complications noted.

## Discussion

We present the first case of homozygous protein C deficiency in the UAE successfully treated with liver transplantation. Although genetic testing for the protein C mutation was not performed in this case due to financial constraints, the patient's clinical presentation, including neonatal purpura fulminans and extremely low protein C levels, strongly suggests homozygous protein C deficiency.

Protein C deficiency is inherited in an autosomal dominant manner. Mutations in a single copy in heterozygous individuals cause mild protein C deficiency, whereas individuals with homozygous mutations present with severe protein C deficiency. Severe protein C deficiency resulting from congenital homozygous mutations presents in neonates soon after birth and characteristically presents as disseminated intravascular coagulation and purpura fulminans [2].

In a case series of 12 Chinese infants, all cases with neonatal purpura fulminans, except one, had a protein C level of less than 10% [3]. A study done in Japan showed that most patients with neonatal onset protein C deficiency had a level of less than 10%; however, late-onset patients had a median protein C level of 31% (range 19-52%). Those infants with double mutations of the protein C gene (PROC) developed purpura fulminans and stroke. They rarely escape infancy and childhood without thrombosis. Individuals heterozygous for PROC are predisposed to thrombosis when infected or distressed [4].

Chakravarty et al. also discussed a case of arterial thrombosis involving the distal aorta, where the parents were first-degree relatives with a history of recurrent fetal losses. The infant’s protein C level was less than 10%, and no genetic test was done [5].

There are few standardized guidelines for treating protein C deficiency, with only a limited number of large studies available. Most of the evidence is derived from case reports and anecdotal experiences. Protein C deficiency can be managed through replacement therapy using protein C concentrate. Neonatal purpura fulminans can be managed with protein C replacement from FFP or viral-inactivated human plasma-derived protein C concentrate [2].

Liver transplantation offers an alternative curative treatment for children with homozygous protein C deficiency. Given the risks of transplant by itself, liver transplantation is considered only in severe cases. The first successful liver transplantation as a curative therapy for congenital protein C deficiency was reported in 1988 by Casella et al., who reported the first orthotopic liver transplantation in a 20-month-old boy with homozygous protein C deficiency. His protein C levels were normalized, and he was free of coagulation disorder symptoms seven months after transplantation. Several reports followed on successful liver transplantation in congenital protein C deficiency [1].

Another study revealed successful liver transplantation for homozygous protein C deficiency using a heterozygous living-related donor. The decision to use this donor was based on multiple factors. Although using a heterozygous donor may result in a slightly elevated lifetime risk of venous thromboembolism, this risk is minimal compared to the high morbidity associated with untreated protein C deficiency. However, most of these events are not life-threatening and typically occur after age 50. A successful liver transplantation in such cases can likely offer the patient many years without the need for aggressive anticoagulation [6].

With an increasing demand for organs, alternatives to deceased-donor liver transplantation have been thought of. Successful living donor liver transplantation was reported in a six-month-old girl with homozygous protein C deficiency who had suffered from frequent thrombotic episodes. Eight years after the transplantation, she remains symptom-free [7].

Furthermore, in 2015, Matsunami et al. reported on the first living domino liver transplantation as a curative therapy for a child with homozygous protein C deficiency using the liver of a patient with maple syrup urine disease [8].

The mother, who is heterozygous for protein C deficiency, was initially considered a potential living donor. However, due to her significant obesity and fatty liver, she was deemed an unsuitable donor. As a result, the patient was placed on the waiting list for a deceased donor liver transplantation.

Possible complications of liver transplantation should be kept in mind. Sakamoto et al. reported that the initial poor graft function was due to hyperammonaemia and coagulopathy, which required continuous hemodiafiltration and infusion of FFP. Liver dysfunction resolved later with normalization of protein C levels on day 22 [9].

## Conclusions

Liver transplantation should be considered as a definitive treatment for children with homozygous protein C deficiency who experience recurrent thrombotic events despite replacement therapy and for those with severely impaired quality of life due to the disease itself. The decision to proceed with transplantation should be made with careful consideration of the patient’s long-term quality of life and the balance between transplant-related risks and the benefits of avoiding continuous anticoagulation therapy.
